# Cardiac Organoids and Gastruloids to Study Physio-Pathological Heart Development

**DOI:** 10.3390/jcdd8120178

**Published:** 2021-12-10

**Authors:** Marisa E. Jaconi, Michel Puceat

**Affiliations:** 1 Faculty of Medicine, Geneva University, 1206 Geneva, Switzerland; 2 Inserm U1251, MMG (Marseille Medical Genetics), Aix Marseille Université, 13885 Marseille, France

**Keywords:** cardiomyocytes, organoids, gastruloids, 3D-culture, morphogenesis, human cardiogenesis, tissue engineering

## Abstract

Ethical issues restrict research on human embryos, therefore calling for in vitro models to study human embryonic development including the formation of the first functional organ, the heart. For the last five years, two major models have been under development, namely the human gastruloids and the cardiac organoids. While the first one mainly recapitulates the gastrulation and is still limited to investigate cardiac development, the second one is becoming more and more helpful to mimic a functional beating heart. The review reports and discusses seminal works in the fields of human gastruloids and cardiac organoids. It further describes technologies which improve the formation of cardiac organoids. Finally, we propose some lines of research towards the building of beating mini-hearts in vitro for more relevant functional studies.

## 1. Introduction

Following fertilization, the human embryo emerges through complex coordinated and time-controlled molecular and morphogenetic events. When the embryo reaches the stage of gastrula, cells have to take major phenotypic decisions in a space- and time-dependent manner to form the three germ layers and later the right organs at the right place [1].

For many years, two hypotheses have been proposed to account for the emergence of shaped tissues and organs within the embryo. In 1969, Wolpert raised the positional theory and proposed that a morphogen which drives the fate of a cell could be sufficient to position it within a tissue [2]. In 1952, Turing had rather proposed that the shape emerged from a self-organizing mechanisms by reaction-diffusion responses to activators and inhibitors [3]. The most recent studies using mammalian embryos and in vitro pluripotent stem cells (PSC) support a role for the crosstalk between tissue shape and cell fate as a determinant of human embryogenesis [1].

Gastrulation is the most important embryonic event in the life of an organism [4]. During this process, cells exhibit important changes in shape while differentiating into specific cell fates. Epithelial-to-mesenchymal transition (EMT) plays a key role in gastrulation as it allows epiblast cells to progress toward the mesoderm and endoderm while ingressing through the primitive streak [4]. Cells that do not ingress acquire the fate of ectodermal cells. The embryonic structure elongates along a precise axis. BMP, WNT and activin A/Nodal pathways, together with mechanical cues, are here the most important pathways and processes which regulate gastrulation and elongation of the embryo.

Early on, it became clear that, as research on human embryo was restricted in time (Day 14) [5], in vitro models would be required to study human embryonic development. Up to recent times, however, it has become challenging to faithfully recapitulate in vitro the complex native environment and in turn different morphogenetic events that temporally shape the human embryo.

For more than 20 years, we have known that human embryonic stem (hES) cells differentiate in 2D in any cell type upon addition of proper growth factors, but do not shape in any embryonic-like structure. The pioneer cell work of the Zandstra laboratory [6] revealed that PSC have an innate property to self-organize by responding to their secreted cues in a position-dependent manner if cultured in a “niche” within a micro-patterned environment. Micro-patterned surfaces have then been extensively engineered for the last decade to better guide the spatial distribution of cells [7]. In 2014, another pioneer study showed how hES cells cultured on micropatterned surfaces and stimulated with morphogens such as BMP4, known as inductors of cardiogenesis [8,9], were capable of self-organisation and formed both the extraembryonic and the three germ layers [10], thus recapitulating gastrulation, and were referred as 2D-gastruloids. Micropatterned areas in fact restrict and constrain the space in 2D, while embryos grow in 3D. Nevertheless, a morphogen-directed differentiation of PSC showed that these cells organized in 3D and formed cardiac-like chambers [11]. Thus, the autonomous self-organizing properties of PSC opened up the field of 3D gastruloids, as well as organ-specific organoids.

**Figure 1:**
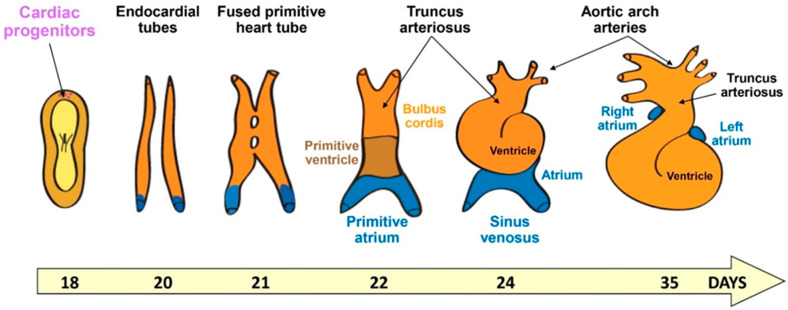
Cartoon illustrating human cardiac developmental stages from gastrulation.

As a ballooned-shaped organ including chambers, the heart is a quite peculiar organ to be engineered in vitro, and grasping the morphogenetic events in 3D is fundamental for it. As shown in Figure 1, the heart forms from the lateral plate mesodermal cells that organize during gastrulation. Indeed, mesodermal cells emerge when epiblast cells ingress through the midline of the embryo between endodermal and ectodermal cells along the long axis (i.e., the primitive streak). The cells then spread laterally and cranially within the embryonic disc. One-week post-fertilization, cardiogenic mesodermal cells move at the cranial border to form a crescent. This crescent composed of both cardiac progenitors and endothelial/endocardial cells will give rise to the cardiac tube. Other cells from the pharyngeal mesoderm posterior to the dorsal wall of the pericardial cavity (i.e., the second heart field) migrate and contribute to both the cranial and caudal regions to elongate the tube. This tube is then segmented into atrial and ventricular components separated by an atrio-ventricular canal and including an outflow tract. The tube will then loop to form the four chambers [12].

This overview presents pioneering studies and challenging research that demonstrate how PSC and their derivatives are capable of recapitulating quite a complex morphogenesis within the recently described 3D gastruloid and organoid models.

## 2. Human Gastruloids to Model Birth of Cardiac Progenitors and Diseases

Martinez-Arias’s laboratory [13] was the first to show that hES cells stimulated with the WNT activating small molecule Chir9901 could form aggregates within a few hours and then elongated along an axis. They observed in two gastruloids that, at one pole, cells express CDX2, a marker of trophoblastic structure, while at the opposite pole, cells express GATA6. Using a reporter line RUES2-GLR, the authors further revealed how hES cells could recapitulate gastrulation when differentiating into Brachyury^+^, SOX17^+^, SOX2^+^ cells, those markers being specific of mesoderm, endoderm and neurectoderm, respectively. These cells localized to specific territories (i.e., layers) and did not mix. Using tomo-sequencing, the authors confirmed, after 72 h of culture, the presence and the spatial organization of the three germ layers. The analysis of spatial gene expression also revealed the presence of cardiac markers from both first (TBX5, HAND1) and second (ISL1, FGF10, MEF2C) heart fields in the anterior region. Unfortunately, the two gastruloids analyzed in the study retracted after 72 h in culture.

A human gastruloid protocol was then improved by Minn et al. [14] using hES cells cultured on micropatterned surfaces of 500 μm diameter. When challenged by BMP4, cells differentiated into the three germ layers, as well as extraembryonic-like and primordial germ cells. Single cell-RNA sequencing confirmed the presence of all cell types with sub-populations of mesodermal cells, including cardiogenic cells.

While cardiac differentiation of pluripotent stem cells in 2D or agregation of PSC-derived myocytes, endothelial cells and fibroblasts within a microtissue lead to maturing cardiomyocytes [15,16,17,18], to date, the human gastruloid model still faces a limitation as, in contrast to mouse gastruloids, no one could yet push forward these gastruloids towards temporally more advanced heart tissues.

However, this limitation could, in principle, be overcome in a near future. Indeed, human PSC are capable of recapitulating the oscillations of the segmentation clock underlying somitogenesis. The clock is regulated by FGF, WNT, Notch and YAP signaling, as observed in the embryo. Gene expression monitored by single-cell RNA sequencing revealed that human PSC in vitro followed a developmental path like that of the mouse in vivo [19]. Thus, PSC are capable of patterning tissue and recapitulating at least the major molecular mechanisms underlying morphogenesis. Even if the formation of the heart is not a linear process, such as somitogenesis, but rather a 3D process regulated by cell-cell interactions, such as endocardial-myocardial cells crosstalk, embryonic cells should position each other, orient their growth, and possibly form the linear tube that may further loop, as in the embryo (Figure 1). Hence, micropatterning may turn out to be helpful in reaching such an organization.

Another open question is that, so far, most publications on human gastruloids used hES cells but not iPS cells, although we doubt that reprogramming could dramatically alter morphogenetic drafts, except for putative epigenetic marks presently unknown. It would therefore be helpful in recapitulating cardiogenesis in patient-specific iPSC-derived gastruloids to uncover molecular or cellular mechanisms underlying morphogenetic defects in the context of cardiac congenital diseases. Hopefully, the state of pluripotency that usually discriminates hES and iPS cells should not affect the formation of iPS cell-derived gastruloids [20].

A recent pionneering study [21] reported an in vitro model for embryo aneuploidy, a situation associated with impaired in vitro fertilisation due to chromosolal instability. To address this issue, they treated gastruloids generated from hES cells on micropatterned circular surfaces with reversine, a small molecule inhibitor of monopolar spindle 1 kinase. They observed that blocking the spindle assembly checkpoint lead to severe chromosome segregation, and, in turn, aneuploidy in many embryonic cells. Moreover, such gastruloids were not capable of forming the germ layers. Interstingly, they could rescue aneuploid gastruloids by adding a few euploids cells [21].

## 3. Human Organoids to Model Cardiac Tissue and Diseases

In recent years, the in vitro generation of 3D cardiac microtissues from human iPS cells has become an increasingly critical procedure for modelling not only the development of healthy human heart tissue [22,23,24], but also heart pathological conditions relevant for disease modelling [18,25,26,27], drug screening [28,29], as well as novel regenerative solutions [30]. In contrast to gastruloids, cardiomyocytes differentiating within organoids featured some electrophysiological and metabolic signs of maturation [18,31]. After a century of cell culture on flat surfaces, scientists are now addressing several challenges of culturing cells in a 3D environment, namely, how to guarantee tissue microarchitecture and space orientation of different cell types, sufficient nutrient feeding, and waste removal. Long-term culture and reproducibility also revealed missing parameters such as resident immune cells in vivo.

### 3.1. Cardiac Organoids: Scaffold or Scaffold-Free?

The aggregation of relevant cells into microtissues called cardiac organoids or cardioids includes the self- or directed-assembly of stem cell-derived cardiac cells or progenitors, fibroblasts, endothelial cells and smooth muscle cells in the presence of extracellular matrix proteins (ECM), whether natural or synthetic, or in scaffold-free conditions, thus relying on the cell intrinsic deposition of ECM. The different relevant cell types separately generated from iPS cells need to be assembled in appropriate ratios to obtain a functional cardiac tissue amenable of reliable electrical, mechanical, and metabolic assessments. Optimal reported cell ratios comprise 70% cardiomyocytes, 15% endothelial cells and 15% cardiac fibroblasts [18,31], or a 10:5.5 ratio when using cardiac progenitors and mesenchymal cells from human iPS cells and endothelial cells from the human umbilical vein [32]. As cell composition in the fetus changes upon development, maturation, and aging, the contribution of cells such as endocardial and epicardial progenitors remains to be temporally addressed. Of particular interest would be the genesis and remodeling of trabeculae leading to tissue compaction, a process regulated by endocardial/myocardial cell interactions and clonal cell growth [33].

**Figure 2:**
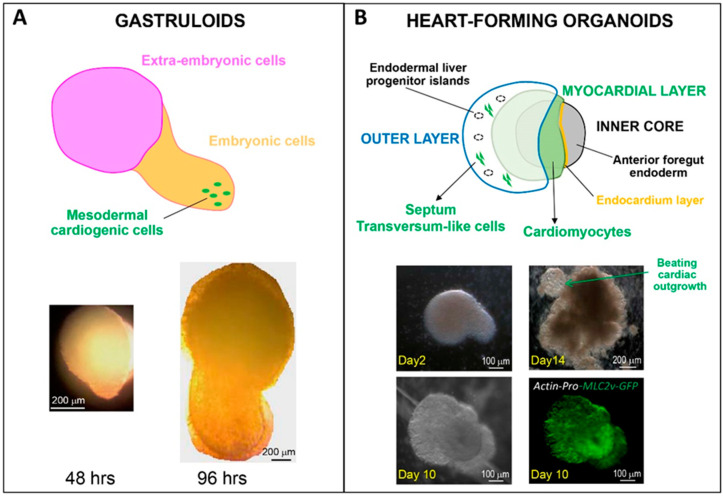
Comparative schemes and microscopy pictures of human PSC-derived gastruloids (**A**) and heart-forming organoids (**B**) (drawing modified from Drakhlis et al. [35]). Organoids were generated from the H9 hES cell line, genetically modified to express MLC2v-GFP under the control of the cardiac acting promoter (**B**).

To date, what are the presently most appropriate protocols to generate organoids that best feature developmental steps of the human heart? From an early developmental perspective, a work from Silva et al.—yet to be peer-reviewed [34]—has analyzed mesendoderm-based spheroids to grasp the role of endoderm–mesoderm interaction in heart formation. Multilineage organoids are particularly interesting to address the spatial contribution of specific cardiac cell sub-types before tube formation. This has been beautifully shown by Drakhlis et al., demonstrating the role of the foregut endoderm [35]. By inducing WNT-dependent cardiac differentiation in self-organizing organoids, they could reproduce the architecture of native heart “anlagen” before heart tube formation. As shown in Figure 2, the multi-layer heart forming organoids were composed of an EC-lined myocardial layer covering an inner core, and surrounded by an outer layer containing endoderm cells, but also cardiac (mainly ventricular) cells from the second heart field and cells of the septum transversum. The whole organoid architecture, however, remained round at early stages and then developed an extrusion containing beating cells.

Recently, however, this germ layer–layer interaction learnt from embryology has been challenged in vitro. Hofbauer and collaborators reported the generation of a mesodermal cell self-organizing cardioid [36]. By challenging hESC with controlled concentrations of WNT activator and activin in the absence of ECM proteins, they observed that mesodermal cells alone could form a chamber-like cavity, otherwise impaired by BMP signaling inhibition, or HAND1 knockout and consequent reduction of Nkx2.5 levels in the cardiac mesoderm. This is a peculiar situation as endodermal tissue, and more specifically the foregut endoderm, is at the origin of the first cardiac cavity formation [37], even if the endoderm and the endocardium are not required for the formation of the bilateral heart tube and of the chamber [38,39], but rather the trabeculae. They further reported that culturing epicardial tissue with the cardioids led to epicardium expansion, migration, and differentiation, as observed in vivo [40]. The role of the epicardium on myocardium expansion in a 3D cardiac microtissue was confirmed by another laboratory [41]. Thus, cardiac organoids have the potential to form chamber-like structures, even if these studies did not consider the left and right ventricle originating from the first and second heart field, respectively.

Further in development is the recent three steps protocol by Lewis-Israeli et al. [42], that is instead based on WNT signaling modulation by chemical inhibitors and added growth factors to produce organoids with internal chambers with organized multi-lineage cardiac cell types. The generation of their organoids required twice as many cells (10,000) as the organoids from Da Silva et al. [34] and Drakhlis et al. [35]. These structures developed complex vasculature and could recapitulate heart field formation and atrioventricular specification, leading to robust functionality. Such a configuration is expected to be particularly suitable to address complex metabolic disorders associated with pathological conditions, such as congenital heart defects, induced, for example, by pregestational diabetes.

Nevertheless, we are still far from obtaining the necessary structural and cellular complexity of the heart tissue in 3D only by self-assembly. As biochemical and biophysical cues govern cellular spatial patterning in tissue morphogenesis and organ formation, many approaches under development are based on patterned substrates to geometrically confine cells under defined mechanical stresses [43]. Interestingly, patterned micromolds endowed with geometric constrains have been shown to drive special organization of human PSC into micro-chambered beating structures [11,44]. Signaling cues such as the WNT/β-catenin pathway can favor self-organizing lineage specification in hES cell colonies to form beating human cardiac microchambers (confined by the pattern geometry), containing cardiomyocytes surrounded by myofibroblasts [11]. This is a clear advantage for studying early aspects of cardiac development such as embryonic spatial patterning and drug-induced developmental toxicity [45]. Yet, organoid size constrains (to limit internal necrosis), throughput efficiency and cell/material waste remain issues to be improved and/or fully addressed to secure R&D screenings, not least in terms of standardization and costs.

### 3.2. 3D Printing Accounting for Human Heart Development: Challenges

Several biomaterials and 3D strategies (not treated in this review) are continuously developed or refined to build coherent prefabricated or bio-polymerized scaffolds capable of improving coherent cell organization, cell–cell communication, survival, and ultimately functional maturation of seeded cells. Ideally, one would like to obtain miniaturized ventricular or atrial tissues containing vasculature and peri- and endocardial layers that can be used for long-term assessments. Although biomechanics, vascularization and scale-up are challenges that remain to be fully solved, exciting advances come from the first reported 3D chambered heart bioprinted with rat cardiomyocytes and ECM to also contain EC-derived blood vessels, by Noor et al. in 2019 [46]. More recently, significant progress was provided by Kupfer et al. who bioengineered a macroscaled human chambered cardiac pump using an ECM-rich printable bioink [47]. An appropriate formulation of integrin-triggering ECM proteins such as Fibronectin and Laminin-111, together with photo-crosslinkable collagen- and gelatin-methacrylate particles, could enable first the proliferation of seeded human iPS cells and then their differentiation in situ toward advanced matured cardiac, endothelial and smooth muscle cells, but not fibroblasts. Such bioprinted and perfusable cardiac chambers (up to 500 μm wall thickness, the highest so far reported) included densely packed functional cardiomyocytes and large vessel conduits of a native heart, and could be maintained for up to six weeks. So far, only few other studies succeeded in measuring pressure-volume dynamics using tissue engineered chambers [48,49].

Based on controlled ECM-integrin signaling, this work underlines the possibility to generate macroscale self-remodeling beating hearts, as occurring during development is very appealing for electrophysiological and pharmacological testing, enabling relevant pressure–volume dynamics studies. Challenges remain as to achieve (*i*) coherent positioning of differentiating pacemaker cells (although electrical pacing could promote connectivity and concerted function), (*ii*) fully cellularized, matured and vascularized constructs, and (*iii*) valve engineering to regulate pressure-controlled emptying and filling (i.e., stroke-volume and preload) within perfusion bioreactors, thus accounting for biomechanic remodeling during development.

### 3.3. Sound-Induced Morphogenesis: A Step Forward in Cardiac Tissue Engineering?

Sounds have been shown to favor, together with micropatterning [7], morphogenesis [50]. Such a technology could be applied to the formation of organoids and/or organoid agglomerations into defined 3D shapes. This is a very appealing new technology that could circumvent many of the drawbacks of 3D printing. Indeed, using acoustic surface standing waves that position cells or organoids at static pressure nodes, it is possible to quickly and spatially orchestrate patterning of different cell types or organoids in space within a hydrogel (reviewed in [50,51,52]). It enabled the engineering of aligned muscle tissue constructs using myoblasts embedded in type I collagen hydrogels [53]. We can foresee the implementation of acoustic node assembly for bottom-up cardiac tissue engineering, thus facilitating the generation of pre-vascularized 3D tissues, as shown for the liver [51]. This technique could nicely overcome the physical constraints due to organoid sphericity.

## 4. Single Cell RNA-Sequencing (RNA-Seq), a Genomic Tool to Monitor Cell Lineages within Cardiac Organoids

Single cell RNA-seq approaches have advanced the gastruloid and organoid fields of research.

Minn et al. [54] used this approach combined with spatial transcriptomics to evaluate the presence of different lineages within gastruloids. The gastruloid formation was initiated by BMP4-treated hESC cultured on a 500 μm patterned disk. First the authors noticed the presence not only of ectodermal, endodermal, mesodermal, and extraembryonic cells, but also of many cells in transitional states. The three germ layers were self-organized, and the pattern of gene expression revealed that gastruloids were reminiscent of 16 dpf monkey early-/mid-gastrulation stages. The mesodermal cells included cells of the primitive, EOMES and BRACHYURY, lateral plate mesoderm expressing PDGFRA, MESP1, APLNR, and HAS2, and cells of the paraxial mesoderm that is positive for TBX6 and DLL3. No cardiac genes were yet detected.

In a more recent publication, the same team looked at the kinetics of gene transcriptional events in 12, 24 and 48 h culture of human BMP4-induced gastruloids. They reported that these gastruloids follow the developmental kinetic of in vivo gastrulation. Epiblast and ectoderm precursors emerged after 12 h, and mesendoderm progenitors were detected by 24 h. These cells give rise to the mesoderm and endoderm by 44 h, The CS7 (Carnegie Stage 7) human gastrula [55] (featuring the same kinetic of gene expression as their in vitro counterparts [14]).

In the Haufbauer et al. paper [36], the authors also used a single cell RNA-seq approach to assess the cell heterogeneity of their cardioids. They found the presence of both myocytes and endothelial cells, the latter featuring an endocardial transcriptomic signature. The respective concentrations of WNT activator and VEGF could determine the ratio of myocardial/endothelial cells. Interestingly, they found endothelial cells with specific expression of genes responsive to mechanosensitive pathways. Although the analysis of the single cell-RNA seq was not well documented, this technology allowed the authors to conclude that the 3D feature of cardioids allowed the cells to differentiate and self-organize as myocardial/endocardial and epicardial cells, as in in vivo.

A few trajectory inference analyses could be applied to the human single cell datasets available so far, as only one study reported such an analysis [14]; more data are thus required. Such further analyses should be very informative, specifically as to the possibility of pushing forward gastruloids in more advanced stages of heart formation.

## 5. Conclusions and Perspectives

**Figure 3:**
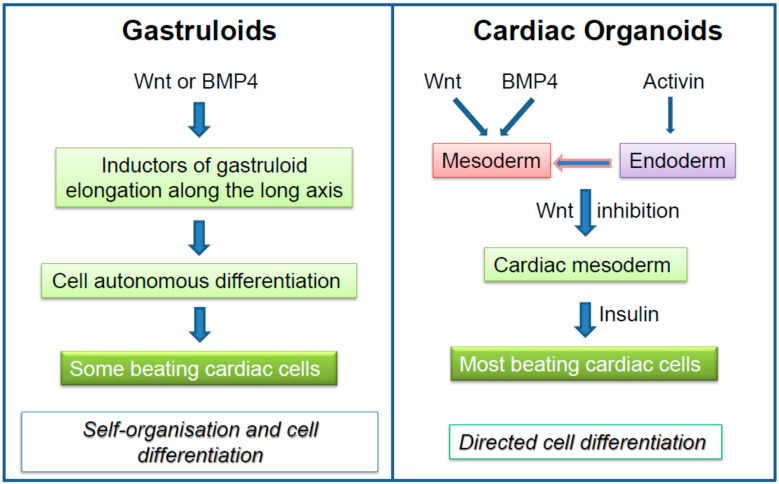
Comparative morphogens and signaling pathways involved in the generation of cardiomyocytes in gastruloids versus cardiac organoids.

One of the main differences between gastruloids and cardiac organoids lies in the major signaling pathways that regulate their development. Figure 3 summarizes the major morphogens that generate cardiomyocytes in gastruloids in a self-autonomous way as compared to morphogen-driven, directed differentiation elicited in cardiac organoids. While cardiac organoids can be maintained in culture for longtime periods, gastruloids are more short-lived. Nevertheless, human gastruloids should further add to the knowledge of human heart formation, but protocols remain to be developed to push forward the gastruloids far beyond the gastrula stage, namely towards the formation of a linear cardiac and then a looped tube.

Manipulating biomechanics coupled to biochemical inputs are key modulatory parameters to induce changes in signal transduction and downstream cellular processes for successful and directed tissue morphogenesis. For the heart, morphogen gradients, lineage tracking, fate mapping in 3D, and spatial transcriptomics (i.e., in situ single cell-sequencing) are critical to dissect heart (micro-)chamber formation. Starting from gastruloid studies, the first proposed bioengineered mini-hearts further underline the necessity of building solid in vitro models of the myocardium at different developmental stages to address causes of cardiac congenital diseases [42,56], and also build efficient drug-screenings [45], in particular via ‘organs on a chip’ platforms (reviewed in [57,58]).

## Data Availability

Not applicable.
